# Mechanism of Longevity Extension of *Caenorhabditis elegans* Induced by *Schizophyllum commune* Fermented Supernatant With Added Radix Puerariae

**DOI:** 10.3389/fnut.2022.847064

**Published:** 2022-03-11

**Authors:** Yongfei Deng, Han Liu, Qian Huang, Lingyun Tu, Lu Hu, Bisheng Zheng, Huaiqing Sun, Dengjun Lu, Chaowan Guo, Lin Zhou

**Affiliations:** ^1^Guangdong Province Key Laboratory for Biotechnology Drug Candidates, School of Biosciences and Biopharmaceutics, Guangdong Pharmaceutical University, Guangzhou, China; ^2^Research and Development Center, Guangdong Marubi Biotechnology Co., Ltd., Guangzhou, China; ^3^School of Light Industry and Food Engineering, Guangxi University, Nanning, China; ^4^School of Food Science and Engineering, South China University of Technology, Guangzhou, China

**Keywords:** anti-aging activity, *Schizophyllum commune*, Radix puerariae, stress resistance, *Caenorhabditis elegans*

## Abstract

*Schizophyllum commune* (*S. commune*) fermented supernatant with added Radix Puerariae (SC-RP) showed significant antioxidant activity in our previous work. However, the possible lifespan and healthspan extending the capacity of *Caenorhabditis elegans* (*C. elegans*) and the underlying mechanism were not illuminated. In this study, the effect of SC-RP on extending the lifespan and improving stress resistance of *C. elegans* were examined. Additionally, the underlying lifespan extending molecular mechanisms of SC-RP were explored. Treated with SC-RP at 10 μg/mL, the lifespan of *C. elegans* increased by 24.89% (*P* < 0.01). Also, SC-RP prolonged the healthspan of the nematode, including reducing lipofuscin levels, improving mobility and enhancing resistance to oxidative stress and heat shock. Moreover, superoxide dismutase and catalase activities were increased for SC-RP treated *C. elegans*. Meantime the intracellular levels of thiobarbituric acid reactive substances (TBARS) and reactive oxygen species (ROS) were attenuated. Express levels of eight genes including *daf-2, daf-16, sod-3, skn-1, gst-4, clk-1, age-1 and mev-1* were analyzed by RT-PCR method for possible *C. elegan* anti-aging mechanisms of SC-RP. Expression levels of key genes *daf-2, gst-4 and sod-3* were up-regulated, while that of *daf-16, skn-1*, and *clk-1* were down-regulated. The results suggest that SC-RP could extend the lifespan and healthspan of *C. elegans* significantly, and the IIS pathway, SKN-1/Nrf2 pathway and mitochondrial metabolism pathway were primarily considered associated. Thus, SC-RP is a potential component to improve aging and aging-related symptoms as new functional materials.

## Introduction

Aging is a physiological phenomenon that occurs in all living organisms, and can lead to behavioral defects and signaling pathway dysfunction such as decreased resistance and physiological decline (1–3). Aging and age-related pathologies are becoming a major global issue because of the aging population (4). Nonetheless, growing evidence has demonstrated that consuming foods rich in polysaccharides, polyphenols and other active compounds from natural sources such as fungi and plants that possess excellent antioxidant properties may protect or reduce the effects of aging on organisms (5–8). Therefore, research on the use of natural active ingredients with excellent antioxidant activity to prevent aging and age-related diseases has received great attention over the last decade.

Recently, fermentation has been applied to extract and enrich the active ingredients in edible plants and fungi and improve their antioxidant/anti-aging capacity (9). As a wellknown edible white-rot fungus, *Schizophyllum commune* (*S. commune*) is popular because this fungus produces exopolysaccharide schizophyllan (SPG), which is used in vaccines and anti-cancer therapies, in oxygen-impermeable films, as an antibacterial hydrogel for food preservation and as an anti-inflammatory agent (10–12). Apart from SPG, other metabolites such as phenolics from *S. commune* may scavenge for free radicals and thus be suitable for use in food and cosmeceuticals industries (13). Furthermore, the fermentation liquid of *S. commune* contains various enzymes, including lignocellulase, xylanase and cellulose (14). A previous study illustrated that *S. commune* can biotransform sophoricoside in *Fructus sophorae* to synthesize new active substances with outstanding toxicity toward MCF-7 cells, thus revealing that *S. commune* has the potential to enrich active ingredients of fermented products and enhance antioxidant, anti-aging or biological properties of phytochemicals in fermentation substrates (15). Therefore, the fermentation system of *S. commune* is a practical platform for improving the biological activity of food or traditional Chinese medicinal materials. Radix Puerariae (RP) is rich in isoflavones, which are primary polyphenolic antioxidants found in medicinal and edible plants (16, 17). Our previous study have confirmed that adding RP to the *S. commune* fermentation system can increase the antioxidant activity of the fermented supernatant, and these antioxidant activities are mainly contributed by the puerarin from RP and some new ingredients synthesized during fermentation such as resveratrol (18). Furthermore, the above results proves the feasibility of *Schizophyllum* liquid fermentation system as a bioreactor and provides a reference for the biotransformation of edible medicinal fungi such as *Cordyceps militaris* and *Ganoderma lucidum* et al.

*Caenorhabditis elegans* (*C. elegans*) has a simple physiological structure, is easily cultured, has a short life cycle, the genetic pathways are well known and there are 60 to 80% of the genomic sequences in *C. elegans* show homology to sequences in the human genome (19). Based on these advantages, *C. elegans* has been used widely to evaluate the anti-aging properties of substances (20–22). In *C. elegans*, signaling pathways regulating aging have been studied extensively, including the insulin/insulin-like growth factor signaling (IIS) pathway, SKN-1/Nrf2 pathway and mitochondrial electron transport chain pathway. These signaling pathways are associated with lifespan and stress resistance (23–25). Research efforts use nematodes as an *in vivo* model to study the anti-aging activities and molecular mechanisms of natural products. Ibe et al. found that fermented soybean extracts can extend the lifespan, provide resistance to oxidation and heat shock and delay lipofuscin accumulation in *C. elegans* (26). Moreover, Shi et al. found that Monascin formed *Monascus*-fermented products enhances oxidative stress resistance *via* regulation of the DAF-16/FOXO-dependent pathway (27). Furthermore, rice kefiran also induces anti-aging effects and upregulates the thermal stress tolerance of *C. elegans* through the IIS pathway by activating the DAF-16 transcription factor (28).

The antioxidant activity *in vitro* of fermented supernatant cultured from *S. commune* in RP supplemented medium was verified (18). However, whether the significant antioxidant activity of SC-RP can play an anti-aging effect remains unclear. In the present study, we used *C. elegans* as an *in vivo* model to systematically survey the longevity effect and mechanisms of action of SC-RP. Our results showed that SC-RP prolonged the lifespan significantly, promoted healthspan behaviors, improved the activities of antioxidant enzymes and enhanced stress resistance of *C. elegans* by regulating multiple mechanisms, including the IIS pathway, SKN-1 pathway and mitochondrial metabolism pathway. These results provide a meaningful understanding of the antioxidant and anti-aging effect of SC-RP, thus facilitating the development and application of SC-RP as a functional food or new pharmaceutical raw material.

## Materials and Methods

### Materials and Reagents

Superoxide dismutase (SOD; A001-2), catalase (CAT; A007-1) and thiobarbituric acid reactive substances (TBARS; A003-1) assay kits were purchased from Nanjing Jiancheng Bioengineering Research Institute (Nanjing, China). 2′, 7′-Dichlorofluorescein diacetate (DCFH-DA) and methyl viologen dichloride (paraquat, 98%) were purchased from Sigma-Aldrich Chemical Co. (St Louis, MO, USA). All other chemicals and solvents were of analytical grade or higher.

### Preparation of SC-RP

The seed medium for *S. commune* 5.43: Glucose 30.0 g, KH_2_PO_4_ 1.0 g, MgSO_4_ 7H_2_O 0.5 g, Yeast extract 3.0 g, and these ingredients were dissolved into 1000 mL deionized water at natural pH (about 6.0). Then sterilized at 121°C for 20 min. The *S. commune* strain was cultured initially in a seed medium as a fermented seed at 28°C for 3 days. The seed culture was inoculated with a 10.0 % inoculum size (v/v) to fermentation medium containing 12.80 g/L RP, and cultivation was carried out on a rotary shaker (ZAZY-78BN, ZHICHU, China) at 160 rpm and 28°C. After 7.0 d fermentation, the fermentation broth was used a centrifuge (GL-21M, CENCE, China) at 15000 g for 10 min to separate the fungi and supernatant, and the SC-RP was sterilized through a 0.22-μm filter membrane and collected for further study (18).

### Determination of Total Phenolic and Flavonoid Contents

Total phenolics were determined by the Folin-Ciocalteu colorimetric method as described previously (29) using gallic acid (GA) as the standard. Total flavonoid content was measured using the sodium borohydride/chloranil method (30), and catechin hydrate (CE) was used as a standard. Data are reported as milligram GA equivalents per 100 g fresh weight (mg GAE/100 g, FW) and milligram CE equivalents per 100 g FW (mg CE/100 g, FW).

### Determination of Polysaccharides and Total Protein Contents

Polysaccharides were determined by the phenol-sulfuric acid method as described previously (31), using glucose as the standard. The reducing sugar content in a sample was determined by the DNS method. On the basis of the manufacturer's instructions, the total protein content was measured using the BCA protein assay kit (A045-4-2; Nanjing Jiancheng Bioengineering Research Institute, Nanjing, China).

### Nematodes Strains and Maintenance

The *C. elegans* strains used in this study were provided by the *Caenorhabditis Genetics* Center (CGC, University of Minnesota, Minneapolis, MN, USA): wild-type Bristol N2, *daf-16* (mgDf50), *age-1* (hx546) I, *mev-1* (zIs356 IV.) II, *clk-1* (e2519) II, *skn-1*(zu135) IV, LD1:ldIs7[*skn-1* b/c::GFP *rol-6 (su100)*], CF1553 {*muIs84* [pAD76 (SOD-3::GFP)]} and TJ356 [*zIs356* IV (p*daf-16-daf-16*::GFP; *rol-6*)]. Worms were raised and maintained at 20°C on nematode growth media (NGM) seeded with *E. coli* OP50 bacteria as a food source starting from the first day of hatching, except where indicated. The SC-RP solutions (1.0 mg/mL in ultrapure water) were diluted into different concentrations by *E. coli* OP50 based on the antioxidant capacity of SC-RP (final concentrations: 5, 10, 20 μg/mL). The utilization of water as a substitute for SC-RP was the control group.

### Lifespan Assay

Age-synchronized N2 nematodes (32) were used for the lifespan assay. The eggs were allowed to hatch on the NGM plates with *E. coli* OP50 until the L4 stage. L4 worms were transferred to NGM Petri dishes containing appropriate working doses of SC-RP (0, 5, 10, and 20 μg/mL). During the reproductive period, worms were scored daily and transferred to new treatment dishes every other day. Worms that failed to move by gentle prodding with a needle were scored as dead. Worms that burrowed into or escaped from the agar or died were removed immediately during the counting process. All assays were performed in three independent trials, and each experimental group included at least 50 worms.

### Reproduction Assay

Age-synchronized L4 stage worms (32) were allowed to lay eggs and transferred to fresh-treated plates every day during the progeny production period. The original plates were stored at 20°C for another day to allow viable eggs to hatch before scoring. The oviposition number of each worm in the breeding time (around 5 to 7 days) was counted, as described by Liu et al. (33).

### Movement Assay

A sinusoidal motion was defined as a one-wavelength shift relative to the long axis of the body (30 individuals in total). The head swing was defined as swinging of the head from one side to the other and then back (30 individuals in total). Locomotivity classes were determined as follows (60 individuals per repetition): the highly mobile worms, which we designated as class A, moved spontaneously and smoothly; members of class B did not move unless prodded and left tracks that were non-sinusoidal; and class C worms did not move forward or backward, but oscillated their nose or tails in response to touch (33). Worms were placed on NGM and allowed to move freely for 1 min before observation.

### Analysis of Lipofuscin Accumulation

Age-synchronized L4 stage Worms (32) were treated with SC-RP as described above to determine the lipofuscin levels. On days 5, 10 and 15 after treated with SC-RP, the worms were placed on 2% (w/v) agarose pads on glass slides and anesthetized with sodium azide (10 mM) (34). The worms were visualized under a fluorescence microscope (MF54-N, Mshot, Guangzhou, China). The fluorescence intensity of the worms was measured using Image J software 8.0 (National Institutes of Health, Bethesda, USA).

### Activities of Superoxide Dismutase (SOD) and Catalase (CAT), and Contents of Thiobarbituric Acid Reactive Substances (TBARS) and Intracellular Reactive Oxygen Species (ROS)

The activities of SOD and CAT and TBARS levels in the supernatant were determined using commercial kits on the basis of the manufacturer's instructions. Endogenous ROS levels in nematodes were measured using a modified DCFH-DA assay (7). In brief, SC-RP-treated worms (~1,000 worms) were crushed by a biological grinder. After centrifugation, the supernatant (50 μL) and DCFH-DA (50 μL) were mixed in a 96-well plate. Fluorescence was immediately quantified by a microplate reader (SPARK 10M, TECAN, Switzerland) at an excitation/emission wavelength of 485/535 nm. Final results were normalized to protein levels obtained using the BCA protein assay kit.

### Stress Resistance Assays

At least 150 Age-synchronized L4 stage worms (32) treated or not treated with SC-RP for 5 days were used to perform three independent biological replicates. Subsequently, worms were exposed to various stresses until all worms died.

H_2_O_2_-induced oxidative stress assay. This assay was performed according to a method described by Lin with minor modifications (6). The worms were transferred to freshly prepared NGM containing 0.1 of 10% H_2_O_2_. The vitality of the worms was examined every 30 min.

Paraquat-induced oxidative stress assay. The assay was performed according to the method reported previously (34). Briefly, survival was monitored every 24 h after the worms were subjected to plates containing 10 mM paraquat.

Heat shock assay. The assay was performed according to the method reported previously (34). The number of dead worms was recorded every hour after the worms were transferred from a 20°C culture environment to a 35°C culture environment.

### Nuclear DAF-16::GFP and SKN-1::GFP Quantitation

For the translocation assay, age-synchronized L4 stage worms (32) pretreated with SC-RP for 5 days at 20°C were transferred onto one microscope slide coated with a 2% (w/v) agarose pad, followed by anesthetization with 10 mM sodium azide for 2 min (33). The worms were observed under a confocal microscope (Nikon Eclipse 80i Microscope SOP, Tokyo, Japan). The distribution of DAF-16::GFP and SKN-1::GFP was identified by the following principles: “cytoplasmic,” “intermediate” and “nuclear,” which represents the percentage in each treated group. All trials were conducted in triplicate using 20–30 worms per group.

### SOD-3::GFP Fluorescence Quantification Assay

CF1553 (SOD-3::GFP) transgenic strains were used to detect the expression of SOD-3. Age-synchronized L4 stage worms (32) were treated with SC-RP for 5 days at 20°C. Subsequently, the worms were transferred onto one microscope slide coated with a 2% (w/v) agarose pad, followed by anesthetization with 10 mM sodium azide for 2 min (34). Images were captured using a fluorescence microscope (MF54-N, Mshot, Guangzhou, China).

### Quantitative Real-Time Polymerase Chain Reaction (qRT-PCR) Assay

Total RNA was isolated from ~1,000 worms (treated for 5 days) by TRIzol® reagent (Invitrogen, Carlsbad, CA, USA) (7), and then these total RNA were synthesized to cDNA using the PrimeScript™ RT Reagent Kit (Takara Biotechnology, Dalian, China). Subsequently, RT-qPCR was conducted using the Bio-Rad Mini Option™ Real-Time PCR Detection System (BioRad, Hercules, CA, USA) with SYBR® Green Supermix fluorescence dye. The expression levels of genes were analyzed using the 2^−ΔΔCt^ method, and β*-actin-1* was the reference gene. The primers used for RT-qPCR are listed in Supplementary Table 1.

### Statistical Analysis

All data were expressed as mean ± SD (*n* = 3). Survival analyses were performed using the Kaplan–Meier method by GraphPad Prism 8.0 software (San Diego, CA, USA), and statistical analyses were conducted using IBM SPSS 25.0 (SPSS Inc., Chicago, IL, USA). Statistical significance was determined by one-way ANOVA with Duncan's multiple comparison post-test, and differences were considered significant at *P* < 0.05.

## Results

### Chemical Composition of SC-RP

As shown in Table 1, the total phenolics and total flavonoids of SC-RP were 3,731.56 ± 54.07 mg GAE/100 g, FW and 27.97 ± 0.41 mg CE/100 g, FW, respectively. SC-RP also contains 6.76 ± 0.47 mg/mL schizophyllan and 4.66 ± 0.05 mg/mL total protein (18). Moreover, the addition of RP introduces puerarin into the fermentation products, with a content of 56.45 ± 3.26 mg/100 g, FW. This result illustrated that fermented supernatant cultured from *S. commune* in RP-supplemented medium is rich in phytochemical components, which may be the main source of anti-aging activity. Therefore, the anti-aging activity of SC-RP was further evaluated.

**Table 1:** Chemical compositions of SC-RP.

**Active substance**	**Contents^**a**^**
Total phenolics (mg GAE/100g, FW)	3,731.56 ± 54.07
Total flavonoids (mg CE/100g, FW)	27.97 ± 0.41
Puerarin (mg/100g, FW)	56.45 ± 3.26
Schizophyllan (mg/mL)	6.76 ± 0.47
Total protein (mg/mL)	4.66 ± 0.05

a *Data were expressed as the mean ± SD (n = 3)*.

### SC-RP Increased the Lifespan of *C. elegans*

**Figure 1:**
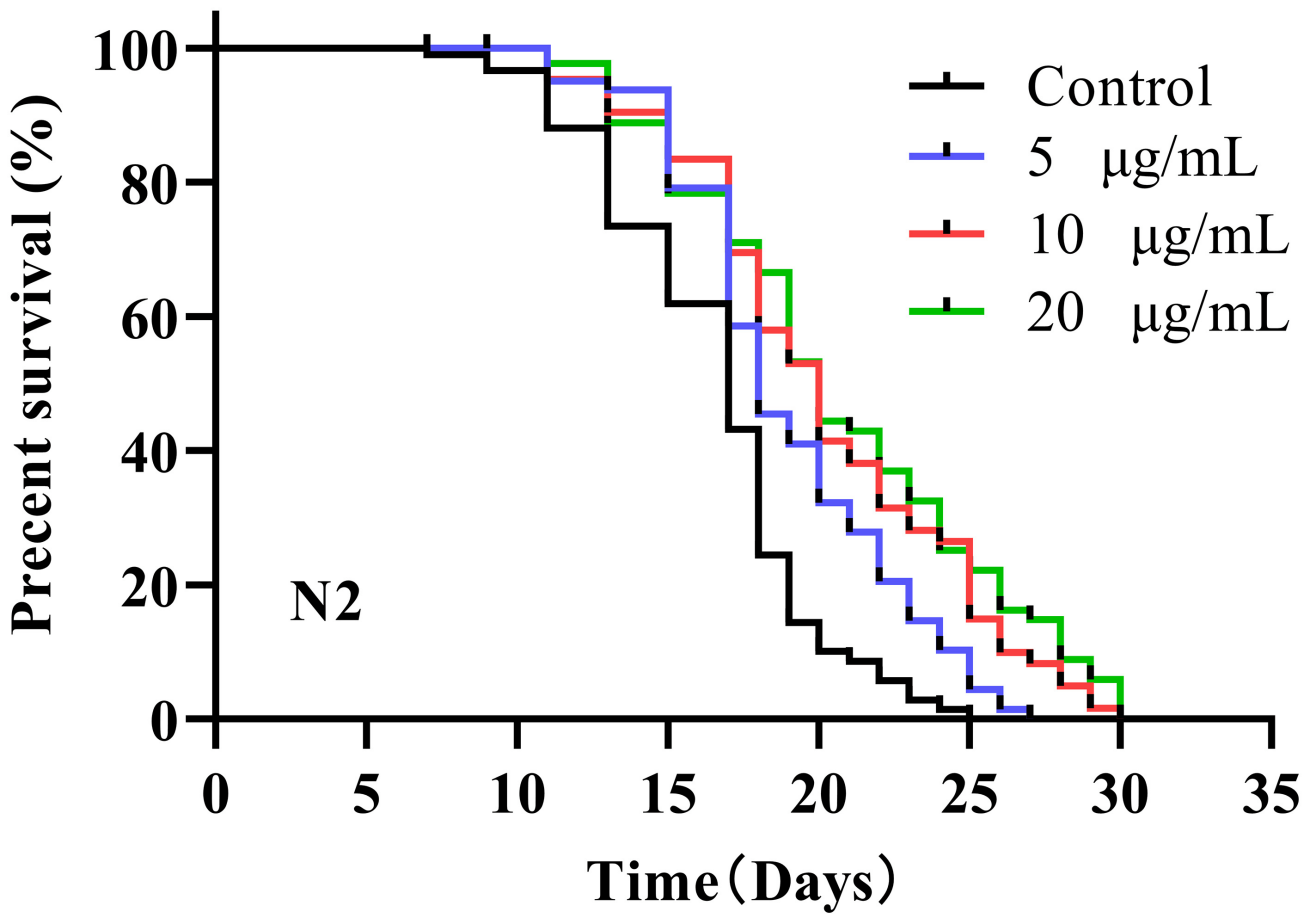
Survival of wild-type *C. elegans* treated with *Schizophyllum commune* fermented supernatant with added Radix Puerariae (SC-RP). *C. elegans* (N2) were treated with final concentrations of 0 (control), 5, 10, or 20 μg/mL SC-RP starting from the L4 stage (day 0). During the reproductive period, worms were scored daily and transferred to new treatment dishes every other day. The survivals were recorded every other day, until all of the worms died. There were at least three independent biological replicates (*n* ≥ 50). The log-rank (Mantel–Cox) test was used for statistical analysis, showing that 5, 10, and 20 μg/mL SC-RP resulted in significant survival curves compared to the control (*P* < 0.001).

A previous study reported that the mean lifespan of wild-type N2 worms was 2–3 weeks at 20°C (7). The average lifespan of N2 control worms under our laboratory conditions was 16.27 ± 1.34 days (Figure 1). After testing 5, 10 and 20 μg/mL SC-RP, the median lifespan of worms increased to 18.88 ± 0.54, 20.32 ± 0.88 and 20.38 ± 1.30 days, respectively, and the corresponding increments compared with the control group were 16.04, 24.89, and 25.26%, respectively (Table 2). This result clearly showed that SC-RP prolongs the lifespan of *C. elegans* significantly at SC-RP doses of 10 and 20 μg/mL (*P* < 0.05).

**Table 2:** Effect of SC-RP on the lifespan of *C. elegans*. (mean ± SD, *n* = 3).

**Group**	**Mean** **Lifespan** **(Days)**	**Maximum** **Lifespan** **(Days)**	**Number**	**Mean fold** **Increase %^**a**^**
Control	16.27 ± 1.34	23.50 ± 2.12	115/150	–
SC-RP (5 μg/mL)	18.88 ± 0.54	26.50 ± 0.71	127/150	16.04
SC-RP (10 μg/mL)	20.32 ± 0.88^**^	30.00 ± 1.00^**^	123/150	24.89
SC-RP (20 μg/mL)	20.38 ± 1.30^*^	31.00 ± 0.00^***^	113/150	25.26

* *P < 0.05;*

** *P < 0.01;*

*** *P < 0.001*.

a *Percentage of mean fold increase is relative to the control*.

### SC-RP Reduced Reproduction Capacity of the Nematode

**Figure 2:**
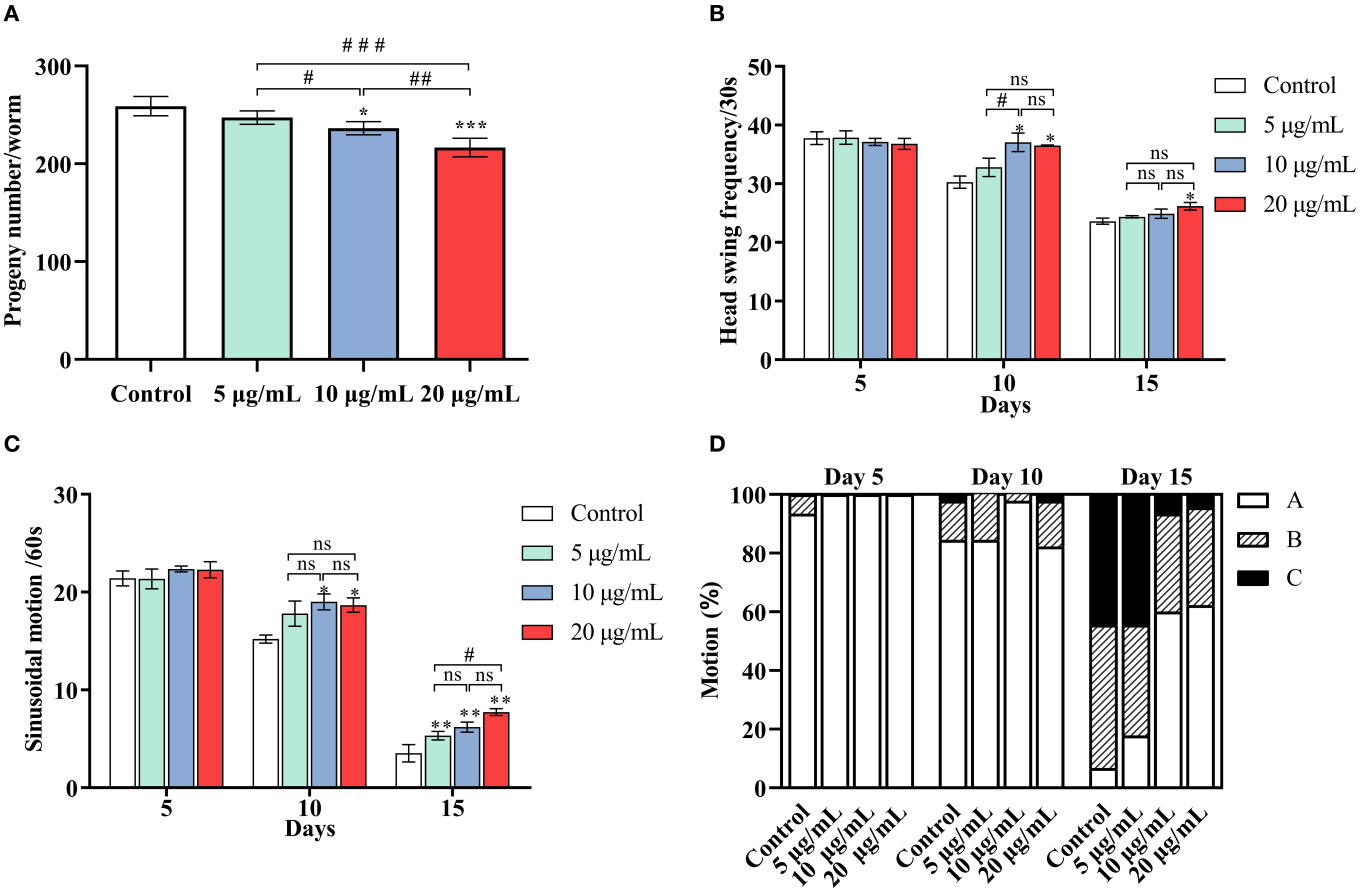
Effect of SC-RP on the physiological functions of *C. elegans*. **(A)** The progeny number of each worm was counted until the parental worms were dead or stopped producing progeny. **(B)** The frequency of the head swing, **(C)** The sinusoidal locomotion, and **(D)** Three levels of locomotivity were performed on days 5, 10, and 15 of the worms. There were at least three independent biological replicates (n ≥ 10 for progeny rate and n ≥ 30 for mobile ability). ^*^P < 0.05, ^**^P < 0.01, and ^***^P < 0.001 indicated statistical significance with control group. ^#^*P* < 0.05, ^##^*P* < 0.01, and ^###^*P* < 0.001 indicated statistical significance in different SC-RP treated groups.

Previous research has shown that life expectancy is positively correlated with a decrease in infertility (35). The number of offspring from each worm was counted to investigate whether the increase in lifespan was accompanied by an improvement in fertility (Figure 2A). The number of offspring per worm in the control group was 259 ± 10 eggs/worm. After administering SC-RP, the total offspring decreased to 247 ± 7 eggs/worm (5 μg/mL group), 236 ± 7 eggs/worm (10 μg/mL group) and 217 ± 10 eggs/worm (20 μg/mL group). These results indicated that SC-RP reduced the fertility of the nematode at doses of 10 and 20 μg/mL and this observation was statistically significant (*P* < 0.05). According to the conservation of energy, it seems plausible that SC-RP prolongs the lifespan and this increase is related to changes in spawning. Nonetheless, extending the lifespan without affecting reproduction remains the most desirable outcome.

### SC-RP Improved the Mobility of the Nematode

Behavioral changes of *C. elegans* and their response to external mechanical stimulation may be associated with the aging process (2). Movement behavior was evaluated using three indicators: head swing (Figure 2B), sinusoidal motion (Figure 2C) and locomotivity (Figure 2D) at the early, mid and mid-late life stages (on days 5, 10, and 15, respectively). In terms of head bend frequency and sinusoidal motion, the worms treated with 20 μg/mL SC-RP exhibited a significant increase in these activities (*P* < 0.05) but 10 μg/mL SC-RP was the most effective concentration on day 10. The decline in motility on day 10 and 15 was markedly delayed in a dose-dependent manner for worms treated with different doses of SC-RP (Figure 2D). Moreover, on day 5, most worms in all groups continued to move freely (class A), and only 6.67% of worms were scored as class B in the control group. On day 10, 84.44% of the worms in the control group were class A, whereas 97.78% of the worms were class A in the groups treated with 10 μg/mL SC-RP. By day 15, SC-RP-treated groups still maintained better motility and presented highly significant dose-dependent differences (*P* < 0.05). The ratio of class A worms administrated with the highest dose was 62.22%, whereas the control group was only 6.64%. Taken together, worms treated with 20 μg/mL SC-RP showed the greatest improvement in mobility and the highest increase in lifespan among the three treatment groups.

### Effects of SC-RP on Lipofuscin Accumulation in *C. elegans* at Different Stages

**Figure 3:**
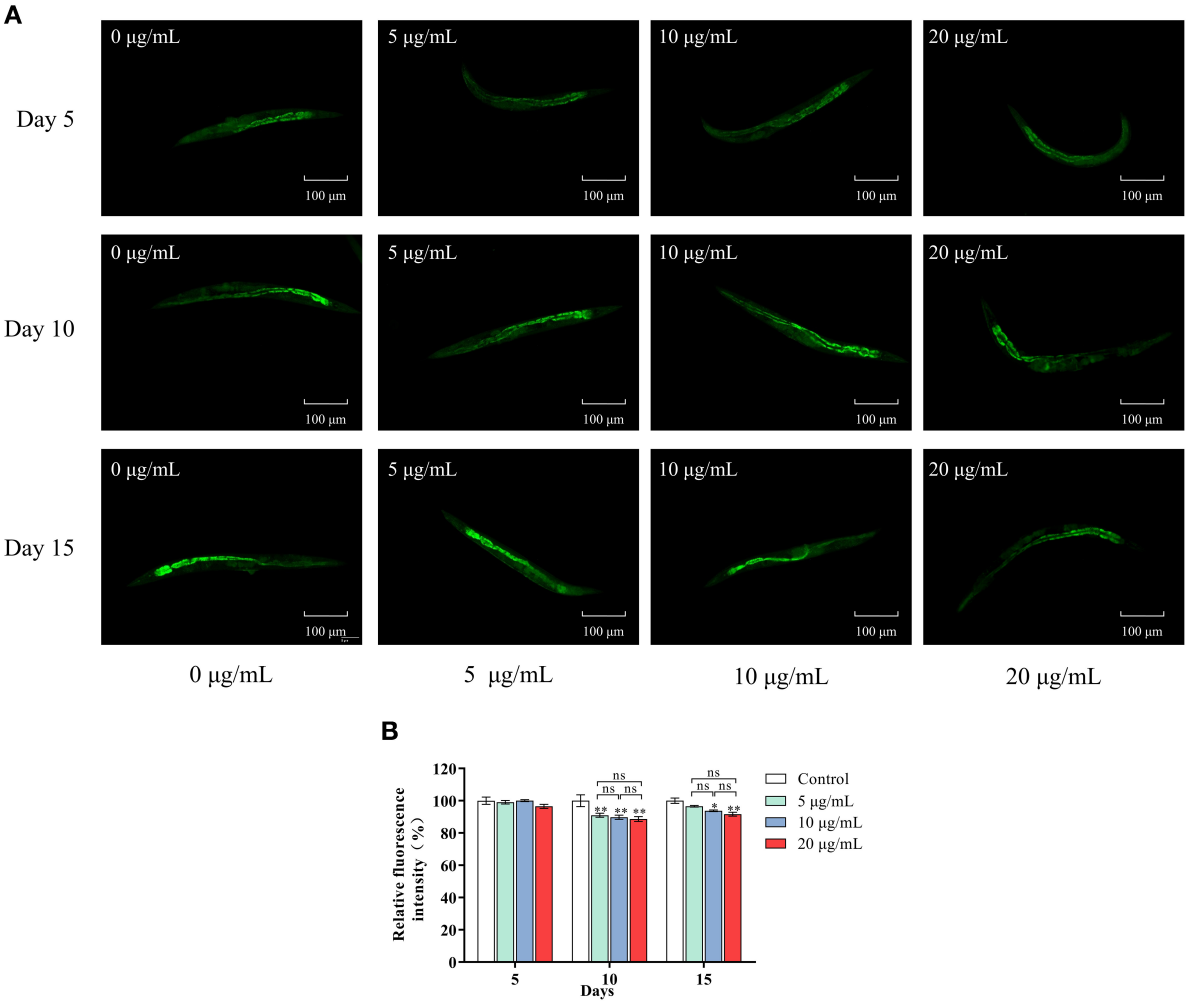
Effect of SC-RP on lipofuscin accumulation in *C. elegans*. **(A)** On days 5, 10 and 15 after hatching, the worms in different SC-RP treated groups were placed on 2% (w/v) agarose pads on glass slides and anesthetized with sodium azide (10 mM). The lipofuscin accumulation of worms was visualized under a fluorescence microscope. **(B)** The relative fluorescence intensity of lipofuscin were quantitated by image J. Data were expressed as the mean ± SD (*n* = 3). ^*^P < 0.05, ^**^P < 0.01, and ^***^P < 0.001 indicated statistical significance with control group.

Lipofuscin accumulation is an essential feature of aging. We determined the effects of SC-RP on lipofuscin accumulation in nematodes by fluorescence intensity measurements (Figure 3). On day 5, no significant difference in age pigment accumulation was observed in all groups, with only a 3.47% reduction in lipofuscin content at high doses of SC-RP. Increasing the SC-RP concentration reduced the accumulation of lipofuscin in nematodes significantly in the mid-late stages (*P* < 0.05), with lipofuscin content in the SC-RP-treated groups decreasing by 7.80% (5 μg/mL group), 10.28% (10 μg/mL group) and 11.37% (20 μg/mL group) when compared with that of the control group on day 10. By day 15, the age pigment accumulation in nematodes for the SC-RP-treated groups still showed a reduction at doses of 10 and 20 μg/mL SC-RP. Moreover, lipofuscin accumulation in worms treated with 20 μg/mL was markedly decreased by 8.36% when compared with that of the control group (*P* < 0.05). Consequently, SC-RP may weaken the accumulation of lipofuscin to improve health indicators.

### SC-RP Rendered *C. elegans* Resistant to Stress

**Figure 4:**
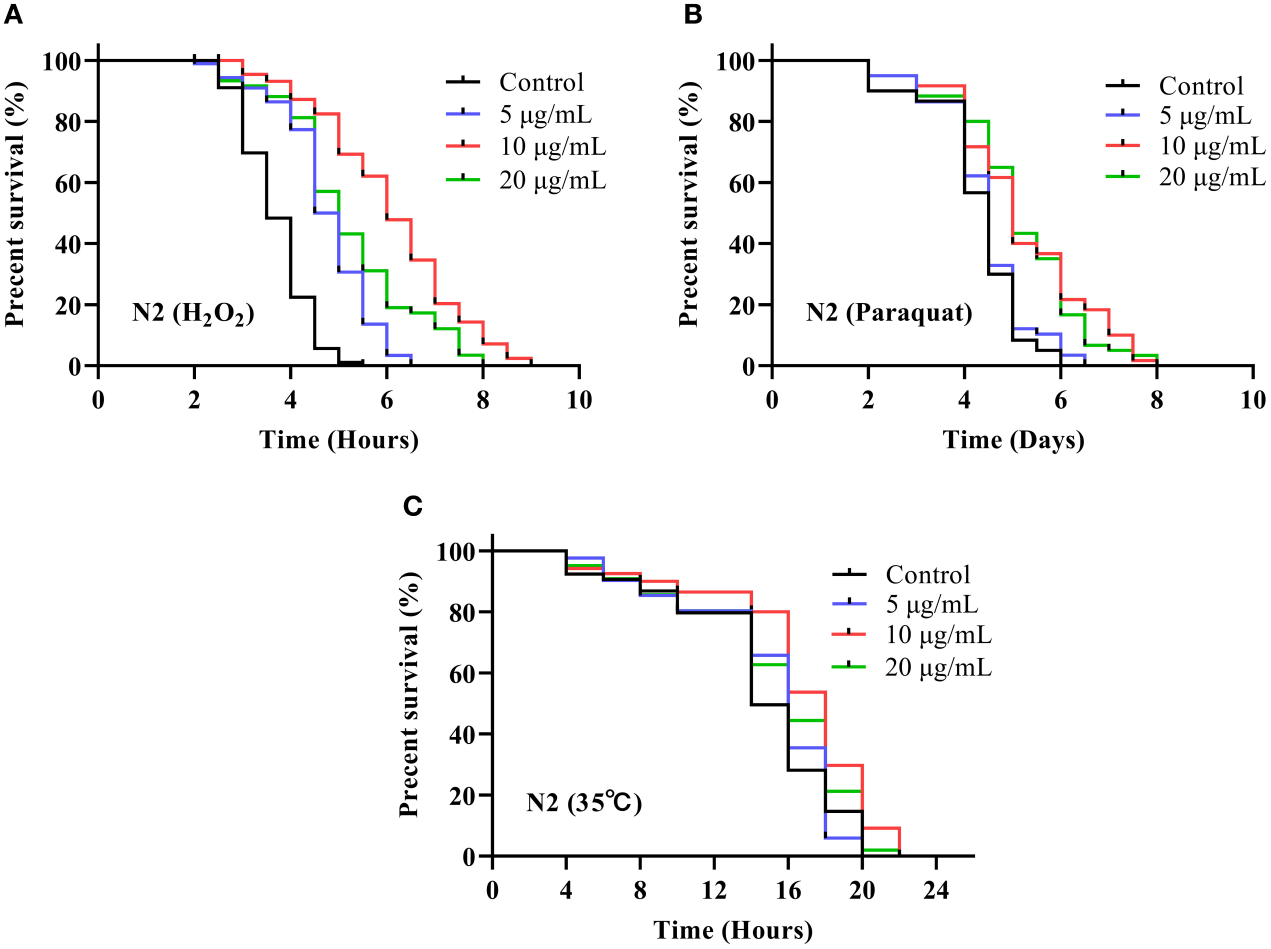
Effect of SC-RP on stress resistance in *C. elegans*. Survival curve of the *C. elegans* N2 strain under **(A)** H_2_O_2_-induced oxidative stress, **(B)** paraquat-induced oxidative stress, and **(C)** heat shock stress. During the reproductive period, worms were scored daily and transferred to new treatment dishes every other day. The survivals were recorded every other day, until all of the worms died. There were at least three independent biological replicates (*n* ≥ 50).

As depicted in Figure 4A, treatment with SC-RP significantly augmented the survival time of N2 worms under H_2_O_2_-induced oxidative conditions when compared with that of the control group. The longest median lifespan was observed for the group treated with 10 μg/mL SC-RP (6.06 ± 0.52 h), which was a significant increase by 65.09% when compared with that of the control group (*P* < 0.01). As depicted in Figure 4B, the mean lifespan of control nematodes was 4.27 ± 0.04 days, whereas those treated with 10 μg/mL SC-RP had a mean lifespan of 5.18 ± 0.32 days, which is a significant increase in the mean lifespan of nematodes by 21.31% (*P* < 0.05). The results indicated that oxidative tolerance was enhanced significantly by SC-RP treatment. Furthermore, the survival time for N2 worms pretreated with 5, 10, and 20 μg/mL SC-RP was increased by 5.53, 14.44, and 7.69%, respectively, when compared with that of the control group in the thermal shock stress test (Figure 4C; Table 3). These results suggest that SC-RP reduced oxidative stress and markedly enhanced the thermotolerance of the nematodes (*P* < 0.05).

**Table 3:** Effect of SC-RP on stress resistance to oxidative stress and thermal shock in *C. elegans* (mean ± SD, *n* = 3).

**Genotype and Condition**	**Treatment**	**Mean** **lifespan**	**Maximum Lifespan**	**Number**	**Mean fold Increase %^**a**^**
N2 (H_2_O_2_, 20°C, Hours)	0 μg/mL	3.67 ± 0.07	5.17 ± 0.29	120/150	–
	5 μg/mL	4.73 ± 0.10	6.33 ± 0.29^**^	118/150	28.89
	10 μg/mL	6.06 ± 0.52^***^	8.50 ± 0.50^***^	114/150	65.12
	20 μg/mL	5.18 ± 0.43^*^	7.75 ± 0.35^***^	116/150	41.14
N2 (paraquat, 20°C, Days)	0 μg/mL	4.27 ± 0.04	5.75 ± 0.35	119/150	–
	5 μg/mL	4.41 ± 0.03	6.50 ± 0.00^**^	118/150	3.28
	10 μg/mL	5.18 ± 0.32^*^	7.75 ± 0.35^***^	120/150	21.31
	20 μg/mL	5.11 ± 0.25^*^	7.75 ± 0.46^***^	120/150	19.67
N2 (35°C, Hours)	0 μg/mL	13.93 ± 0.80	20.00 ± 0.00	112/150	–
	5 μg/mL	14.70 ± 1.18	20.00 ± 1.36	116/150	5.53
	10 μg/mL	15.93 ± 0.89^**^	22.00 ± 0.46^**^	119/150	14.44
	20 μg/mL	15.00 ± 0.54	21.33 ± 1.15	113/150	7.69

* *P < 0.05;*

** *P < 0.01;*

*** *P < 0.001*.

a *Percentage of mean fold increase is relative to the control*.

### SC-RP Increased Antioxidant Enzyme Activities and Decreased the Accumulation of TBARS and ROS

**Figure 5:**
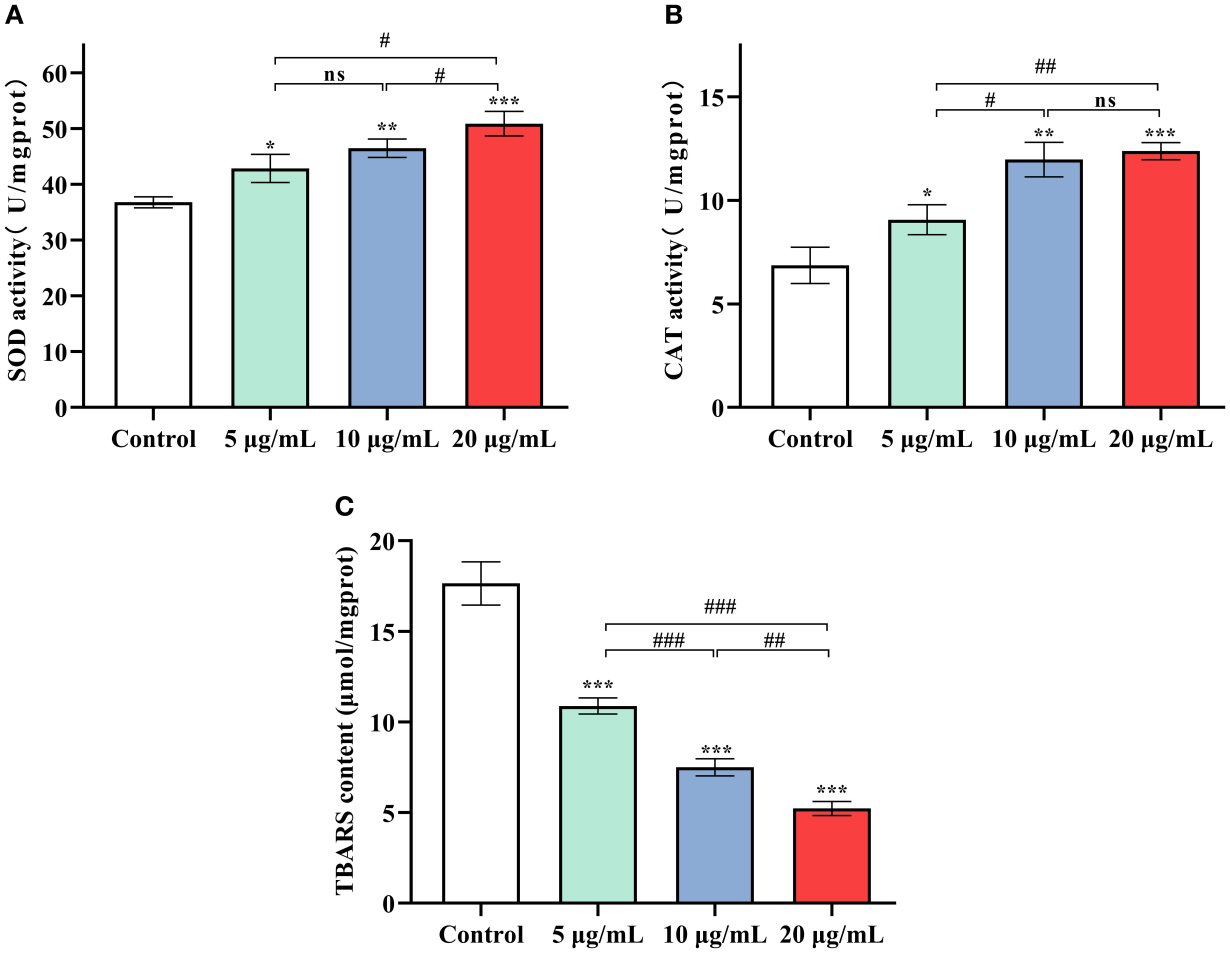
Effect of SC-RP on the activities of antioxidant enzymes SOD **(A)** and CAT **(B)**, and the level of TBARS **(C)** in *C. elegans*. The activities of SOD and CAT and TBARS levels in the supernatant were determined using commercial kits on the basis of the manufacturer's instructions. Final results were normalized to protein levels obtained using the BCA protein assay kit. Data were expressed as the mean ± SD (*n* = 3). ^*^P < 0.05, ^**^P < 0.01, and ^***^P < 0.001 indicated statistical significance with control group. ^#^*P* < 0.05, ^##^*P* < 0.01, and ^###^*P* < 0.001 indicated statistical significance in different SC-RP treated groups.

**Figure 6:**
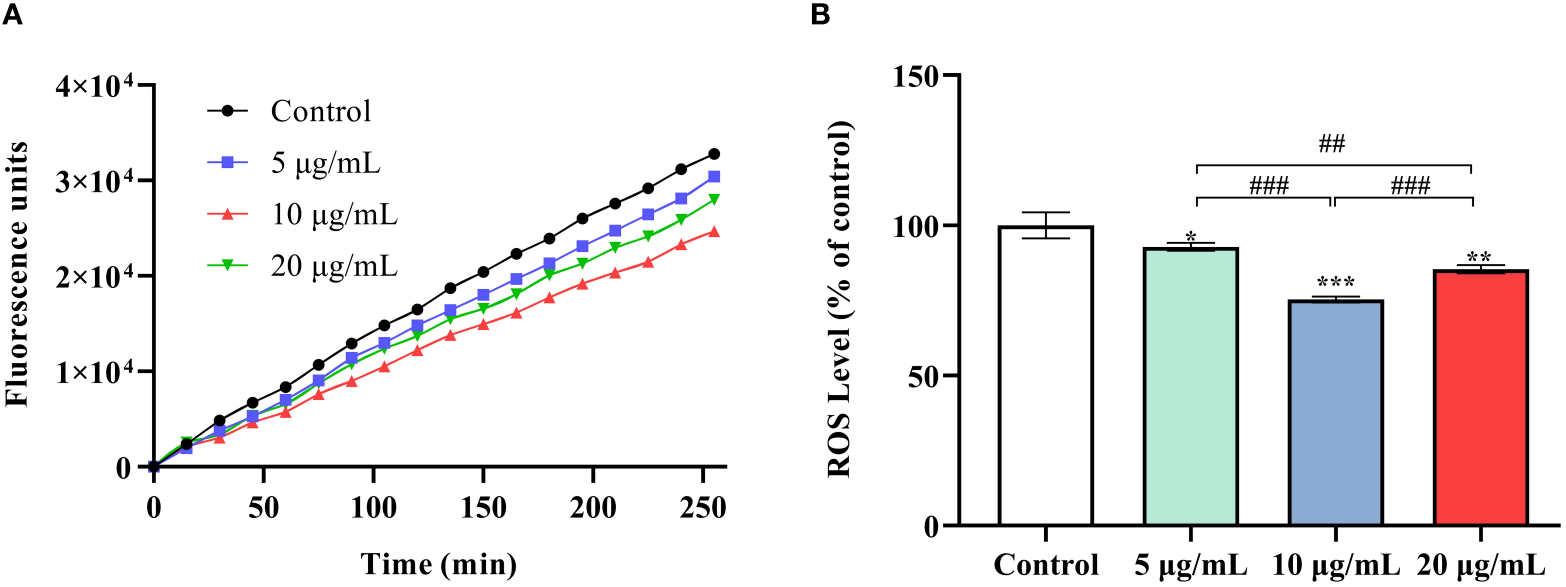
Effect of SC-RP on the oxidative stress sensitivity of wild-type N2 worms. Relative formation of ROS after 96 h of exposure to SC-RP (0, 5, 10, or 20 μg/mL). SC-RP-treated worms (~1000 worms) were crushed by a biological grinder. Endogenous ROS levels in nematodes were measured using a modified DCFH-DA assay. **(A)** The curve showed the accumulation of the fluorescence intensity of ROS in the nematodes. **(B)** The ROS accumulation level in nematodes. Final results were normalized to protein levels obtained using the BCA protein assay kit. Values are means ± SD (*n* = 3). ^*^P < 0.05, ^**^P < 0.01, and ^***^P < 0.001 indicated statistical significance with control group. ^#^*P* < 0.05, ^##^*P* < 0.01, and ^###^*P* < 0.001 indicated statistical significance in different SC-RP treated groups.

At 5, 10 and 20 μg/mL SC-RP, SOD activity in worms was observed to increase significantly by 16.56, 26.38, and 38.33%, respectively (Figure 5A, *P* < 0.05). The same tendency was also observed for CAT activity (Figure 5B). The high dose (20 μg/mL) SC-RP treatment group resulted in an 80.42% increase in CAT activity when compared with that of the control group (*P* < 0.001). In addition, the TBARS content in the high dose (20 μg/mL) group was reduced by 70.39% when compared with that of the control group (*P* < 0.001). Furthermore, the ROS level was reduced by 6.71, 25.76, and 13.56% in the 5, 10, and 20 μg/mL SC-RP treatment groups, respectively, when compared with that of the control group (Figure 6, *P* < 0.05). Clearly, SC-RP showed significant antioxidant potential by enhancing the activities of SOD and CAT and reducing the levels of TBARS and intracellular ROS.

### SC-RP Regulated the Insulin/IGF-1 Signaling (IIS) Pathway

**Figure 7:**
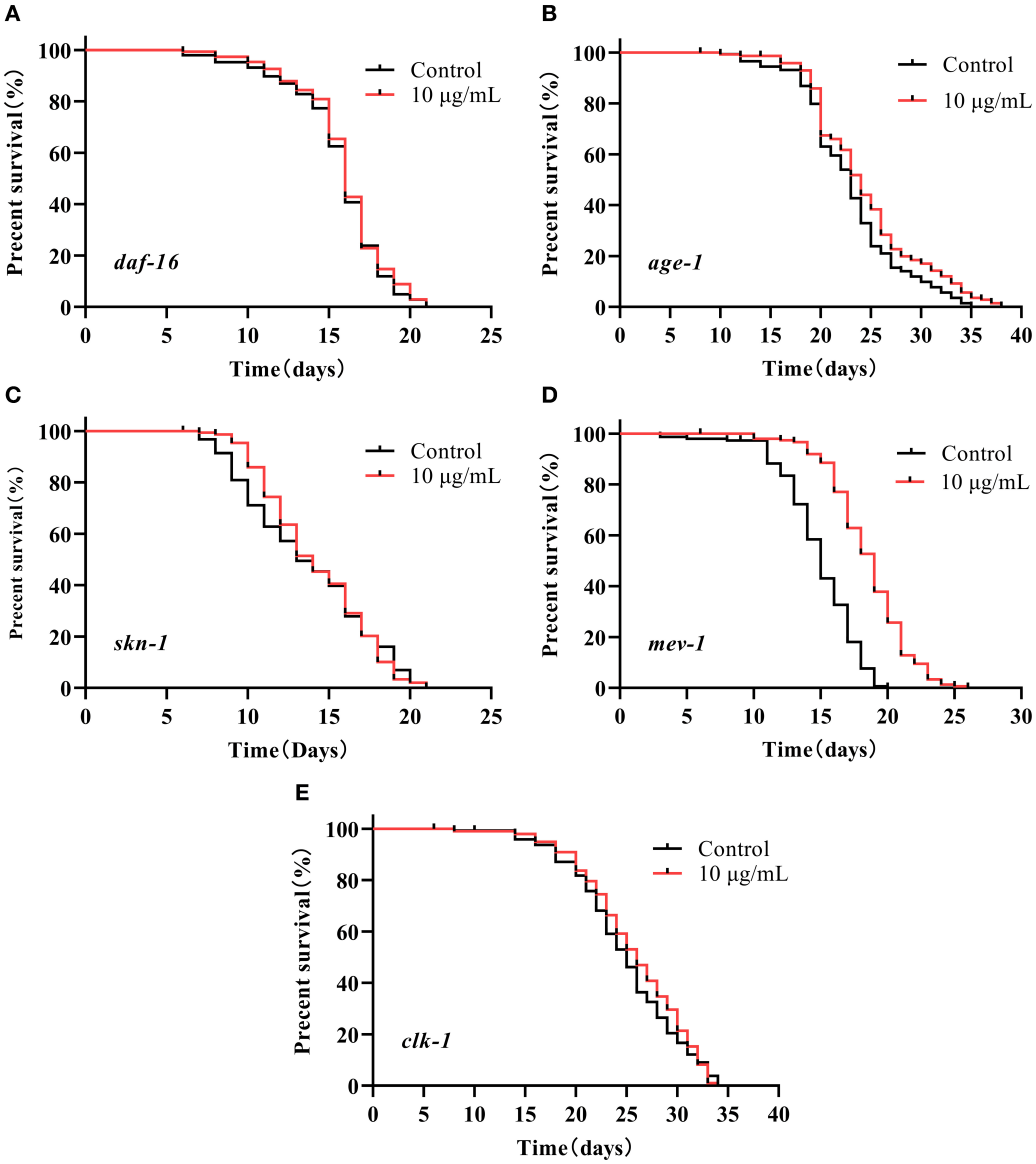
Survival of several mutant strains treated with SC-RP (representative results). **(A–E)** Loss-of-function mutant strains in several genes related to lifespan regulation were treated with final concentrations of 0 (control) or 10 μg/mL SC-RP starting from the L4 stage (day 0). The survivals were recorded until all of the mutants died (*n* = 134–150 worms/treatment). The log-rank (Mantel–Cox) test was used for statistical analysis.

**Figure 8:**
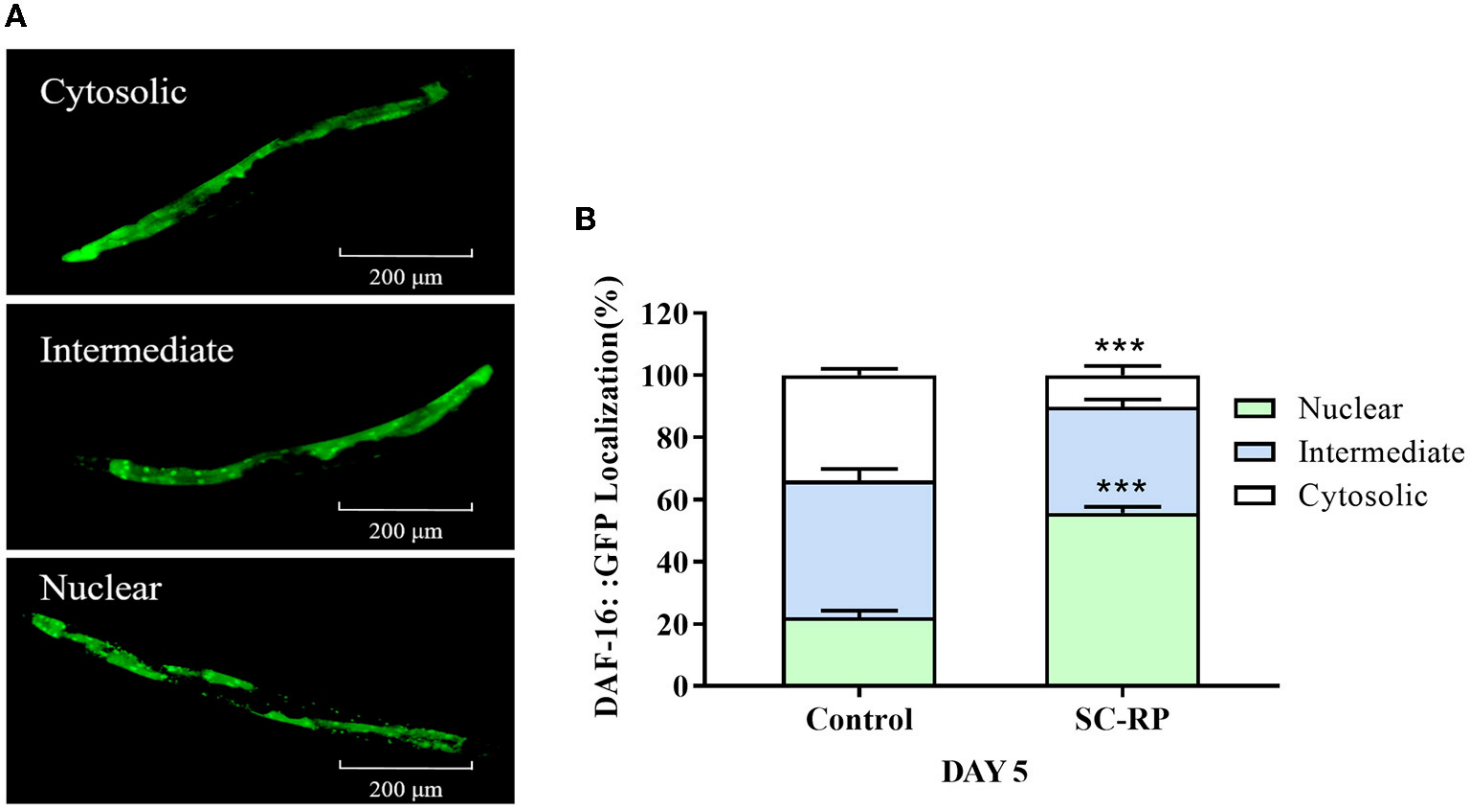
Effect of SC-RP on subcellular localization of DAF-16. **(A)** Representative fluorescence images of TJ356 worms with cytosolic (top), intermediate (middle), and nuclear (bottom) DAF-16::GFP localization. **(B)** The number of transgenic TJ356 worms of each category was measured (*N* > 20). Data were expressed as the mean ± SD (*n* = 3). ^*^P < 0.05, ^**^P < 0.01, and ^***^P < 0.001 indicated statistical significance with control group.

We investigated the lifespan of *daf-16* mutant and upstream gene *age-1* mutant to assess whether the IIS pathway was associated with extending the lifespan of SC-RP-treated nematodes. We found that 10 μg/mL SC-RP treatment did not extend the lifespan of *daf-16* mutant but prolonged the lifespan of *age-1* mutant (Figures 7A,B; Table 4), demonstrating that the IIS pathway is required and the *daf-16* gene may play a role in increasing the lifespan of nematodes treated with SC-RP. In addition, the localization of DAF-16 in nuclei is an essential prerequisite for activating target gene transcription by this protein. We next used the TJ356 strain containing a GFP::DAF-16 transgene to further determine the effect of SC-RP on DAF-16 subcellular localization. As shown in Figure 8A, the TJ356 green fluorescent protein subcellar distribution can be divided into cytosolic (top), intermediate (middle) and nuclear (bottom). The results showed that the nuclear fraction increased from (22.11 ± 3.65) to (55.76 ± 3.23)%, whereas the cytosolic location of DAF-16 reduced from (33.95 ± 3.54) to (10.15± 1.19)% (Figure 8B). This finding suggested that SC-RP may directly activate DAF-16 or molecules located upstream of DAF-16.

**Table 4:** Effect of SC-RP on the lifespan of *daf-16 (mgDf50)* mutants, *age-1 (hx546)* mutants*, clk-1(e2519)* mutants*, mev-1(kn1)* mutants, and *skn-1(zu135)* mutants (mean ± SD, *n* = 3).

**Genotype and condition**	**Treatment**	**Means** **lifespan**	**Maximum lifespan**	**Number**	**Mean fold increase%/** **Genetic requirement^**a**^**
*daf-16 (mgDf50)* (20°C, Days)	0 μg/mL	15.56 ± 0.18	20.33 ± 0.71	147/150	yes
	10 μg/mL	15.84 ± 0.05	20.67 ± 0.58	150/150	
*age-1 (hx546)* (20°C, Days)	0 μg/mL	22.98 ± 0.57	34.33 ± 1.15	150/150	-
	10 μg/mL	24.47 ± 0.56^*^	37.67 ± 0.54^***^	147/150	6.48
*clk-1(e2519)* (20°C, Days)	0 μg/mL	24.90 ± 0.06	34.00 ± 0.00	134/150	yes
	10 μg/mL	24.88 ± 0.12	32.33 ± 0.58^*^	147/150	
*mev-1(kn1)* (20°C, Days)	0 μg/mL	14.89 ± 0.26	19.33 ± 0.58	145/150	-
	10 μg/mL	18.55 ± 0.56^***^	25.00 ± 1.00^***^	148/150	24.58
*skn-1(zu135)* (20°C, Days)	0 μg/mL	14.49 ± 0.72	21.00 ± 0.00	141/150	yes
	10 μg/mL	15.01 ± 0.50	20.67 ± 0.58	146/150	

* *P < 0.05;*

*** *P < 0.001*.

a *Percentage of mean fold increase is relative to the control*.

**Figure 9:**
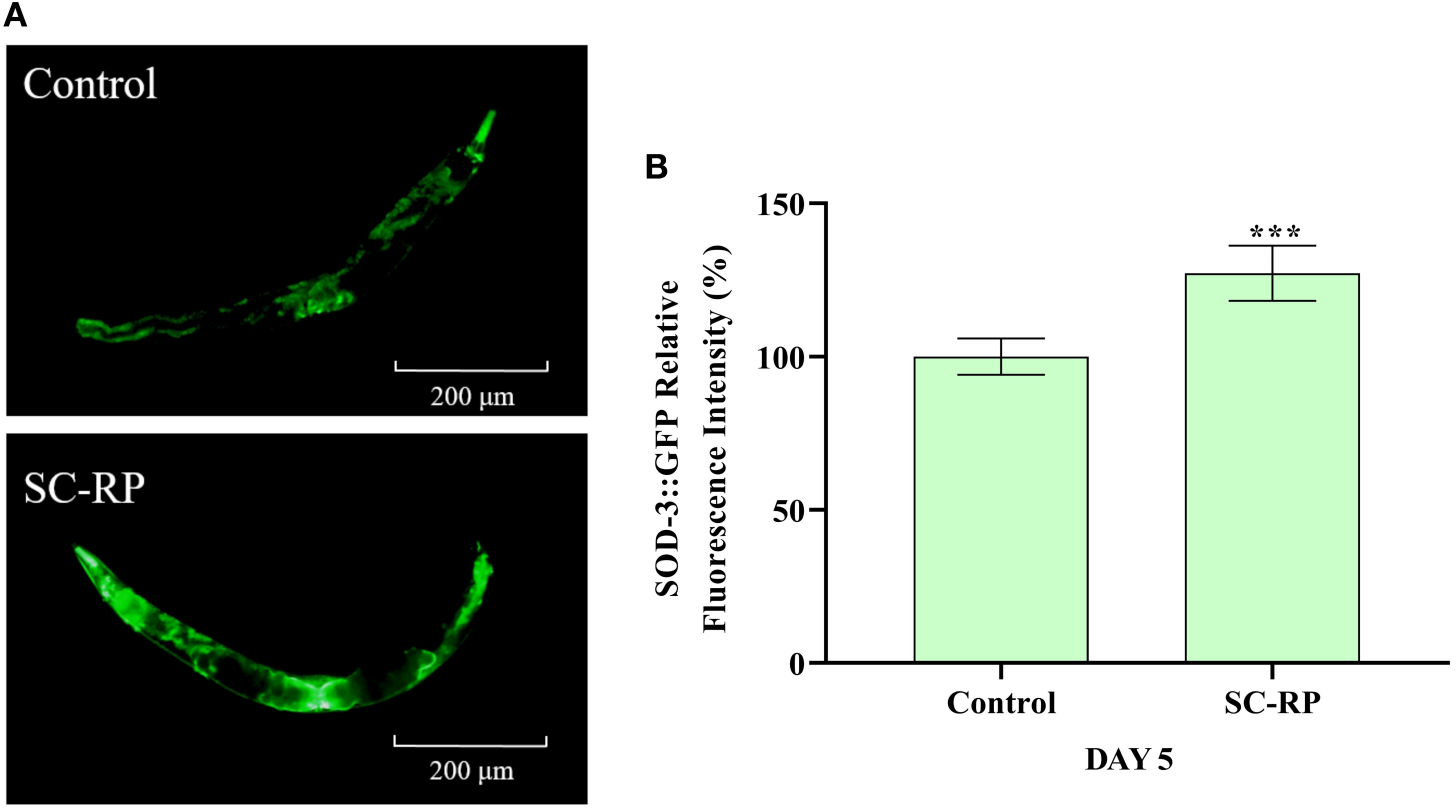
Effect of SC-RP on the relative fluorescence intensity of SOD-3::GFP **(A,B)**. The relative fluorescence intensity of SOD-3::GFP was quantified using Image J software (*N* > 20). Data were expressed as the mean ± SD (*n* = 3). ^*^P < 0.05, ^**^P < 0.01, and ^***^P < 0.001 indicated statistical significance with control group.

The expression levels of the *daf-16* gene and its upstream/downstream genes were examined to explore the underlying anti-aging mechanisms of SC-RP further. After treatment with 10 μg/mL SC-RP, the relative expression level of *daf-16* decreased to 0.12-fold, and the levels of *age-1, daf-2 and sod-3* increased by 1.08 ± 0.03, 6.24 ± 0.92, and 4.47 ± 0.04 times, respectively (**Figure 11A**). Moreover, we also observed significant expression in the mean fluorescence intensity of *SOD-3*p::GFP following SC-RP treatment (Figures 9A,B, *P* < 0.001), which further indicated that SC-RP may reduce oxidative stress by regulating the expression of *daf-16-*related genes. Taken together, these results indicated that SC-RP extended the healthspan of *C. elegans* through modulation of the IIS signaling pathway.

### SC-RP Regulated the SKN-1/Nrf2 Pathway

The decrease in the activity of the IIS signaling pathway directly promotes SKN-1 nuclear accumulation in the intestine and activates downstream target genes, which is parallel to DAF-16 activity. A lifespan assay of the *skn-1* (zu135) mutant was performed to determine whether SKN-1 affects the healthspan of SC-RP-treated nematodes. Treatment with 10 μg/mL SC-RP showed no effect in prolonging the lifespan in the *skn-1* mutant, indicating that SC-RP extends the lifespan of the nematode in a *skn-1*-independent manner (Figure 7C; Table 4).

**Figure 10:**
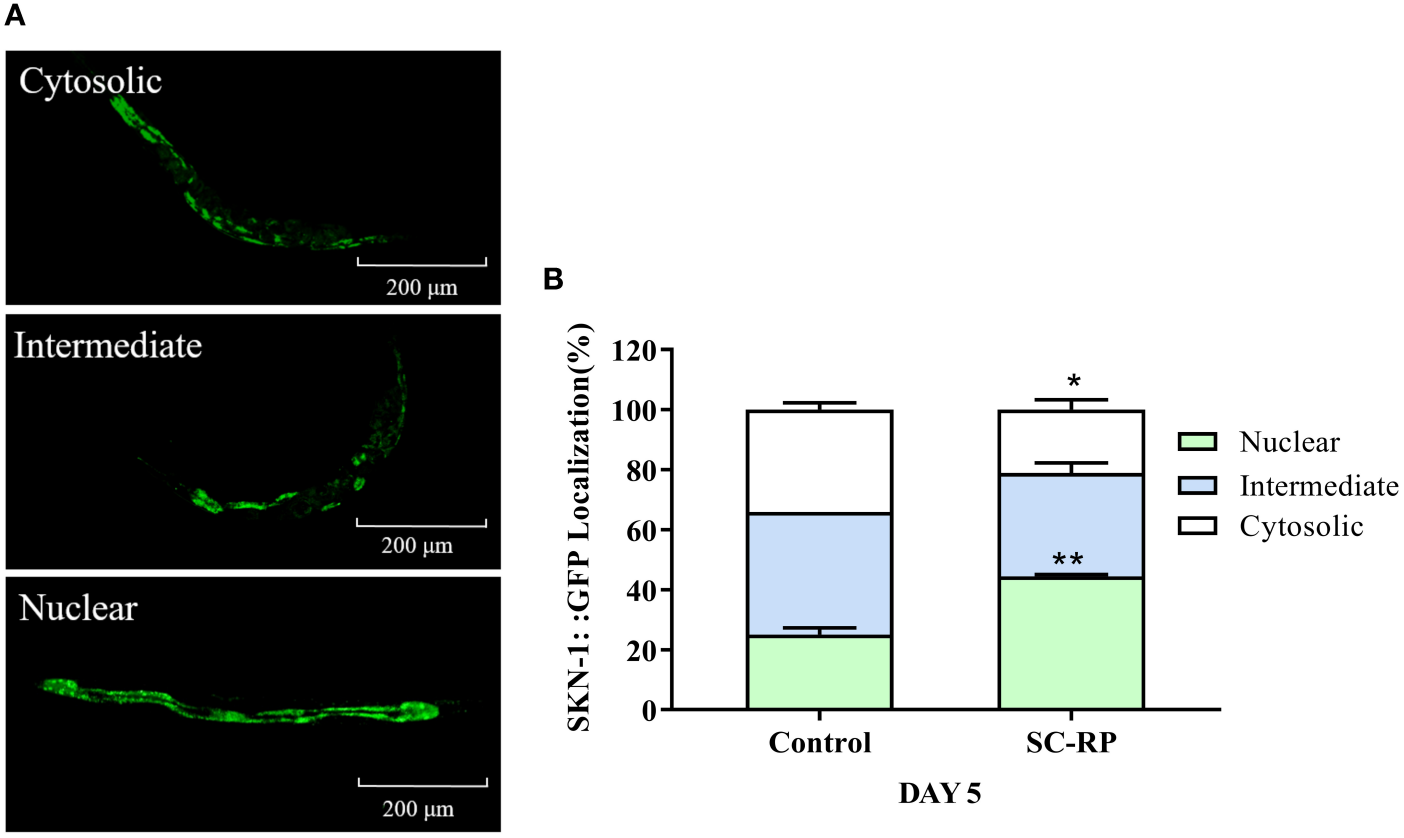
Effect of SC-RP on subcellular localization of SKN-1. **(A)** Representative fluorescence images of SKN-1 worms with cytosolic (top), intermediate (middle), and nuclear (bottom) SKN-1:: GFP localization. **(B)** The number of transgenic SKN-1 worms of each category was measured (N > 20). Data were expressed as the mean ± SD (*n* = 3). ^*^P < 0.05, ^**^P < 0.01, and ^***^P < 0.001 indicated statistical significance with control group.

**Figure 11:**
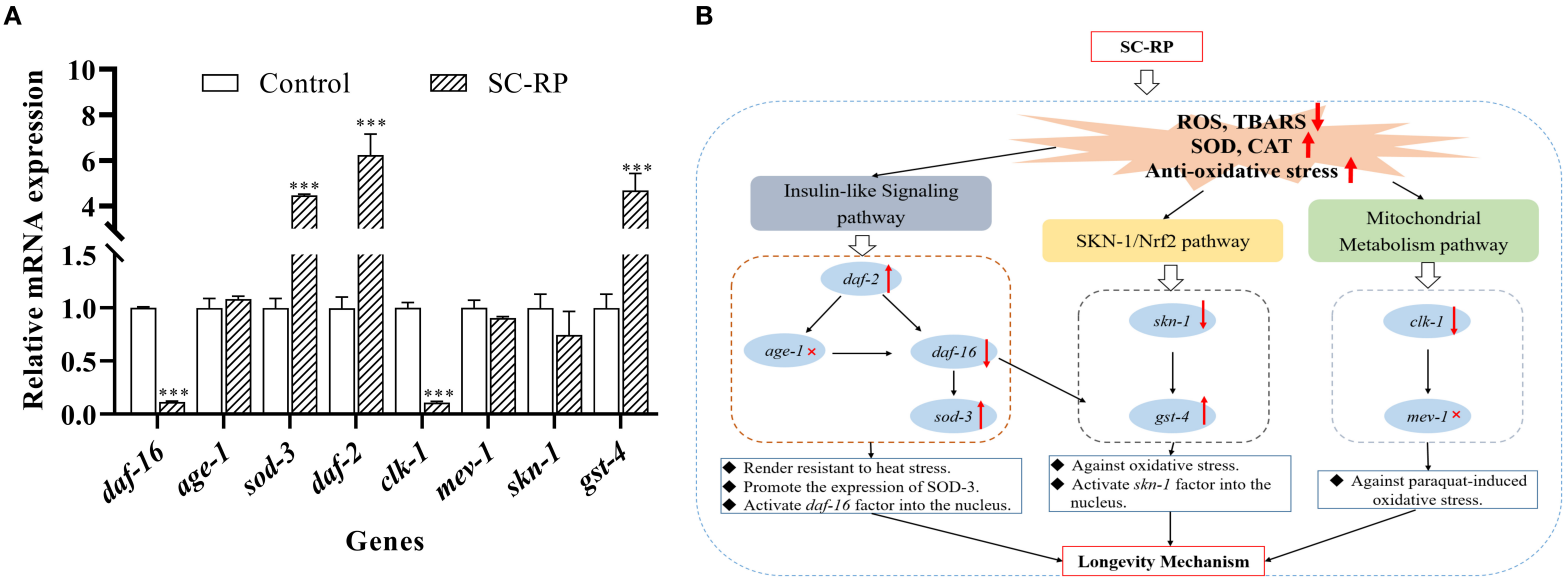
Molecular mechanism of SC-RP in the anti-aging effect. **(A)** The mRNA levels of *daf-16, age-1, sod-3, daf-2, skn-1, gst-4, clk-1, and mev-1* were determined by quantitative real-time RT-PCR and normalized to the expression of β*-actin-1*. **(B)** The up and down arrows respectively represent the up and down expression of genes, and these gene-related pathways play important roles in the regulation of aging. The gene with a red cross was showed no relation to the SC-RP's regulation of aging by measuring the lifespan of mutant nematodes and the aging-related gene expression of nematodes. The above results reveal the possible mode of action for the longevity extension mediated by SC-RP. Data were expressed as the mean ± SD (*n* = 3). ^*^P < 0.05, ^**^P < 0.01, and ^***^P < 0.001 indicated statistical significance with control group.

The localization of SKN-1::GFP was measured using the transgenic strain LD1 to study further the role of SKN-1 in extending the nematode lifespan (Figures 10A,B). The results showed that nuclear translocation of SKN-1 in the intestine increased from (25.00 ± 3.21) to (44.46 ± 0.99)% and cytosolic localization of SKN-1 decreased from (34.09 ± 3.21) to (21.12 ± 5.78)% (Figure 10B), indicating that SC-RP promotes translocation of SKN-1 into the nucleus significantly. In addition, we explored the effect of SC-RP on the expression of *skn-1* and its downstream gene glutathione-S transferase-4 (*gst-4*). The results showed that compared with the control group, the mRNA expression level of *skn-1* decreased to 0.63 ± 0.07-fold, and the expression level of *gst-4* increased by 4.68 ± 0.75-fold (Figure 11A), suggesting that SKN-1 may play an essential role in regulating the lifespan of nematodes treated with SC-RP.

### SC-RP Regulated the Mitochondrial Electron Transport Chain Pathway

Oxidative stress is a key factor of aging and ROS is primarily generated from mitochondrial metabolism (36). However, whether SC-RP extends the lifespan through the mitochondrial electron transport chain pathway is unknown. Thus, the anti-aging effect of SC-RP on *mev-1* and *clk-1* worms was evaluated. SC-RP treatment was observed to extend the mean survival time by 24.6% in *mev-1* mutant, demonstrating that SC-RP improves survival during mild oxidative stress (Figure 7D; Table 4). We then measured the *clk-1* mutant lifespan, and the results revealed no increase in the median lifespan, demonstrating that SC-RP may act independently of *clk-1* (Figure 7E; Table 4). Moreover, quantitative real-time PCR results showed that the mRNA level of *mev-1* and *clk-1* was reduced by 0.90 ± 0.01- and 0.11 ± 0.02-fold, respectively (Figure 11A).

In summary, SC-RP treatment was dependent on CLK-1 activity but acted independently of MEV-1. Thus, the mitochondrial electron transport chain is partially associated with extending the lifespan of SC-RP-treated nematodes.

## Discussion

Numerous studies have shown that compounds and extracts derived from edible plants containing polysaccharides and polyphenols can extend the lifespan and healthspan of a variety of species (6, 37). In our previous research, RP acted as the exogenous additive in a submerged culture of *S. Commune*, with the resulting fermented supernatant (SC-RP) containing a considerable phytochemical composition (18). The phenolic content of SC-RP was 8- to 18-times higher when compared with that of a blueberry extract (38), which suggests that SC-RP is a potentially abundant source of phytochemicals. In this study, we initially found that fermented supernatant cultured from an RP-supplemented medium extends the lifespan and healthspan of *C. elegans*. And, we also founded that SC-RP are not dose-dependent to exert an antiaging effect, which may highly have related to the SPG's higher viscosity to binds tightly to phenolics and flavonoids. Notably, a dose of 10 μg/mL SC-RP prolonged the mean lifespan of *C. elegans* (by 24.89%) to a greater extent when compared with different kinds of fermented products (Figure 1; Table 2) (26, 28) and polysaccharides from *notoginseng* (5), and even reached comparable levels of activity observed previously in crude extracts of edible plants such as a *Rhodiola* extract and the synergistic effects of apple peel and blueberry extract (37, 39). In contrast to the mycelial water extract (MWE) from fermented mycelia of *Cordyceps sobolifera* (40). SC-RP also markedly prolonged the mean survival time of *C. elegans* by ~24%. Furthermore, we found that SC-RP exhibited a similar effect on extending the lifespan of *C. elegans* when compared with that of MWE when the working dose (10 μg/mL) is only one percent of MWE (1 mg/mL), demonstrating that biotransformation can efficiently enrich active substances. Moreover, various research efforts have confirmed that the biological activity of plants and herbal medicines can be enhanced by fermentation because of the biotransformation interactions among multiple phytochemicals and microorganisms (41, 42). Therefore, we propose that the magnitude of the mean lifespan extension of *C. elegans* treated with SC-RP rather than with a single composition may be attributed to interactions among various compounds such as puerarin, SPG and some phenolics or flavonoids in SC-RP. Characterization of these interactions requires further investigation. In addition, SC-RP contains various active ingredients listed above, it is possible the SC-RP extended the lifespan *via* restricting bacterial growth. To verify this idea, we cultured *E. coli* OP50 in LB medium with SC-RP at a working solution (5, 10, and 20 μg/mL). And SC-RP did not inhibit the growth of *E. coli* OP50 in any bacterial growth (data not shown). Therefore, the SC-RP did not extend the lifespan through antimicrobial effect. Besides, low doses of SC-RP (5 μg/mL) did not extend the lifespan of nematodes (Table 2). Alive E. coli *OP50* has poor metabolic capacity at 20°C, thus the influence of killed- E. coli *OP50* or alive E. coli *OP50* to SC-RP was not significant in our test.

Intracellular ROS imbalance can cause aging, which may cause many chronic diseases such as coronary heart disease, osteoporosis, and diabetes (4, 43, 44). The free radical theory of aging proposes that with aging, the accumulation of ROS exceeds the clearance rate by the body, thereby disrupting the redox balance of cells, which leads to the accumulation of oxidative stress and accelerates the aging process (45). Endogenous oxidative stress represents the main factor of aging. Stress in nematodes affects signaling pathways and related genes that play a crucial role in regulating lifespan and various diseases. Various studies have shown that extending *C. elegans* lifespan is associated with enhancing and improving stress resistance (7, 46). In this study, *C. elegans* exhibited increased tolerance against abiotic stress (heat shock) and oxidative stress after supplementing with SC-RP (Figure 4; Table 3). As byproducts of aerobic metabolism, ROS can damage the body. Our previous results showed that SC-RP exhibits good antioxidant activity at the chemical and cellular level when compared with fermented supernatant cultured from a regular medium (SC) at the same concentration of SPG (data not shown), which may be related to puerarin and other active substances present in SC-RP. The present results showed that SC-RP may exert oxidative stress resistance and anti-aging effects by increasing the antioxidant activity *in vivo*. Our present results showed that SC-RP noticeably reduces the ROS and TBARS levels and promotes the activities of antioxidant enzymes such as SOD and CAT (Figures 5, 6), suggesting that the antioxidant activity of SC-RP may contribute strongly to its anti-aging effect. In addition, we found that SC and SPG do not extend the lifespan of *C. elegans* (Supplementary Figure 2; Supplementary Table 2). Therefore, we hypothesize that components of RP such as puerarin and substances produced by biotransformation like phenolics and flavonoids play a key role in the anti-aging effect and characterizing the role of these compounds will be the focus of future research efforts.

Our results revealed that SC-RP improved both healthspan and lifespan. Thus, the effect of SC-RP on the transcriptional expression of key genes and mutants was examined. In *C. elegans*, many genetic manipulations and pathways affect aging, including insulin/IGF-1 signaling, SKN-1/Nrf2 signaling and the mitochondrial respiration pathway (24, 33, 47). The IIS pathway is linked to diet, metabolism, growth, development, longevity and behavior in *C. elegans*. Inhibition of the insulin/IGF receptor DAF-2 reduces the activity of the phosphoinositide 3-kinase (PI3K)/Akt kinase cascade, which, in turn, dephosphorylates and activates the transcription factor DAF-16/FOXO by enhancing DAF-16 translocation from the cytoplasm to the nucleus, where it controls the expression of various genes that contribute to stress resistance and longevity (48). Nematodes with reduced IIS pathway activity are resistant to heat and oxidative stress, indicating that increasing the prevention or repair of oxidative damage can extend the lifespan of *C. elegans* (28, 49). In the current study, SC-RP prolonged the lifespan of *C. elegans via* activation of the insulin/IGF-1 pathway by regulating *daf-16* and *daf-2* but not *age-1* (Figures 7A,B, 11A; Table 4). Moreover, oxidative stress may inhibit IIS-induced phosphorylation and promote the translocation of DAF-16 into the nucleus. As SC-RP extended the lifespan of *C. elegans via daf-16* (Figures 7A, 8; Table 4), it may reduce insulin-like signaling, thus activating DAF-16 and promoting the transcriptional activation of genes targeted by DAF-16. A previous study reported that the lifespan extension and DAF-16 translocation coincide may not necessarily be causally connected (50). Therefore, we inferred that the inconsistent behavior of *daf-16* gene expression and translocation in this experiment may be influenced by upstream related factors thus forming a negative feedback regulation and also occurred in *skn-1* gene in our research. In addition, we confirmed the upregulation of *sod-3* expression by qRT-PCR, which plays an important role in oxidative stress and regulating the transcription activity of DAF-16 (Figure 9; Figure 11A).

Therefore, we conclude that the longevity effect of SC-RP in *C. elegans* involves the IIS pathway.

Activation of SKN-1 is also vital in mediating longevity and antioxidant activities in *C. elegans*. SKN-1 of *C. elegans* is the functional ortholog of the mammalian transcription factor Nrf2, which accumulates in the intestinal nucleus and activation expression of phase 2 detoxification genes such as *gst-4* in response to stress. Similar to the IIS pathway, *skn-1* is involved in various genetic and pharmacologic interventions and thus promotes longevity of *C. elegans* (47). The current study indicated that SC-RP promoted the downregulation of *skn-1* expression and abolished the extension of the lifespan of *skn-1 (zu135)* mutant (Figure 7C; Table 4). Furthermore, SC-RP promoted nuclear translocation of SKN-1 into the nucleus and *gst-4* expression (Figure 11A). These findings suggest that SKN-1 plays a key role in extending the lifespan of SC-RP-treated *C. elegans*.

Mitochondrial respiration plays a major role in energy production and mediates the aging process. ROS are the main byproducts generated from mitochondrial metabolism, which can damage mitochondria and thus affect the lifespan of *C. elegans* (51). Thus, we evaluated the possible role of mitochondrial function in extending the lifespan and the anti-aging effect by studying *mev-1* and *clk-1* worms. The *mev-1* strain produces above-average levels of ROS in mitochondria because of incomplete reduction of O_2_ in the electron transporter chain, which indirectly leads to a reduced lifespan of the worm (52). *clk-1* encodes an evolutionarily conserved enzyme required for the biosynthesis of ubiquinone (UQ; co-enzyme Q; CoQ). The long-lived *clk-1* mutant lacks the endogenous form of coenzyme Q10, which carries electrons and protons from mitochondrial complexes I and II to complex III (53). We illustrated that SC-RP extends the life span of the *mev-1* mutant but did not affect the lifespan of the *clk-1* mutant (Figures 7D,E; Table 4). Moreover, SC-RP downregulated the expression of *clk-1* significantly, showing that SC-RP may function in a *clk-1*-dependent manner but is independent of *mev-1* for extending the longevity of *C. elegans*.

In summary, insulin/IGF-1 signaling, SKN-1/Nrf2 and mitochondrial metabolism pathways were affected by SC-RP treatment and therefore are partly responsible for extending the healthspan of *C. elegans*. The current findings indicated that SC-RP enhances the expression of *daf-2, sod-3* and *gst-4* genes and downregulates the expression of *daf-16, skn-1* and *clk-1* genes (Figure 11A). Therefore, we propose that the longevity effect of SC-RP involves the IIS pathway, SKN-1/Nrf-2 pathway and mitochondrial metabolism pathway (Figure 11B). Moreover, the specific regulation pathway of SC-RP will be verified in future work.

## Conclusions

The current study showed that SC-RP synergistically decreased age-related behaviors and increased the mean lifespan of *C. elegans*, and improved the resistance of *C. elegans* to stress. The lifespan of nematodes treated with SC-RP was extended by 24.89% when compared with that of non-treated (control) nematodes (*P* < 0.01). SC-RP also markedly promoted the activities of antioxidant enzymes (SOD and CAT) and reduced TBARS and ROS levels in cells (*P* < 0.05), indicating that SC-RP contributes to the anti-aging effect by enhancing the activity of antioxidant enzymes. Moreover, mutant assays illustrated that SC-RP-mediated life extension is related to *daf-16, skn-1* and *clk-1* expression levels. SC-RP was found to extend longevity by upregulation of *daf-2, sod-3* and *gst-4* expression and downregulation of *daf-16* and *clk-1* expression. In addition, SC-RP stimulated the migration of DAF-16 and SKN-1 into the nucleus and increased SOD-3 activity. Our finding indicated that the IIS pathway, SKN-1/Nrf-2 pathway and mitochondrial metabolism pathway are associated with the longevity afforded by SC-RP. The present findings provide a scientific basis for using SC-RP as an anti-aging agent, and SC-RP may be suitable for developing functional foods and nutraceuticals products. This study also provides a reference for biotransformation and application of edible medicinal fungi such as *Cordyceps militaris* and *Ganoderma lucidum* et al.

## Data Availability Statement

The original contributions presented in the study are included in the article/Supplementary Material, further inquiries can be directed to the corresponding author/s.

## Author Contributions

YD responsible for the methodology, data curation, formal analysis, investigation, and draft of the manuscript. HL responsible for methodology and software and validation. QH: analysis summary and discussion. LT and LH: assisted testing. BZ and HS: formal analysis. DL and CG: formal analysis and supervision. LZ: organized, supervised, and provided overall guidance of the project. All authors contributed to the article and approved the submitted version.

## Funding

The authors are grateful for the financial support from the Guangdong Pharmaceutical University Research Project (43255098), Marubi Joint Research Project Fund (YB202109131043), and Guangzhou Science and Technology Plan Project Fund (202103000055).

## Conflict of Interest

YD and LT are graduate students jointly trained by Guangxi University and Guangdong Pharmaceutical University. CG, LH, HS, and HL is employed by Guangdong Marubi Biotechnology Co., Ltd, and the experimental part of this research was completed in the R&D laboratory of Guangdong Marubi Biotechnology Co., Ltd. The remaining authors declare that the research was conducted in the absence of any commercial or financial relationships that could be construed as a potential conflict of interest.

## Publisher's Note

All claims expressed in this article are solely those of the authors and do not necessarily represent those of their affiliated organizations, or those of the publisher, the editors and the reviewers. Any product that may be evaluated in this article, or claim that may be made by its manufacturer, is not guaranteed or endorsed by the publisher.
