# Supplementary material for: Mechanism of Longevity Extension of Caenorhabditis elegans Induced by Schizophyllum commune Fermented Supernatant With Added Radix Puerariae
Source: Front Nutr. 2022 Mar 11;9:847064. doi: 10.3389/fnut.2022.847064 (PMC8963188; doi:10.3389/fnut.2022.847064)
Supplement: Supplementary file 1 [file Table_1.pdf]

SUPPLEMENTS

Figure S1.

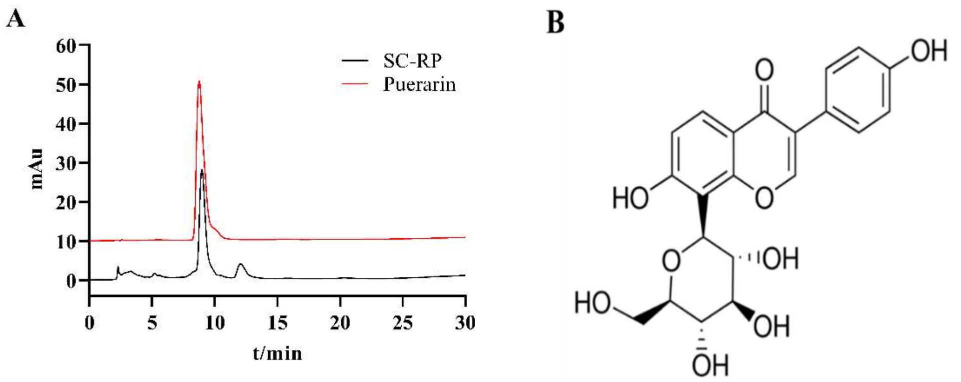

Figure S1. (A) HPLC chromatogram of SC-RP and the reference standards of (1S)-1, 5-Anhydro-1-[7-hydroxy-3-(4-hydroxyphenyl)-4-oxo-4H-chromen-8-yl]-D-glucitol (Puerarin), (B) The structure of the Puerarin.

Figure S2.

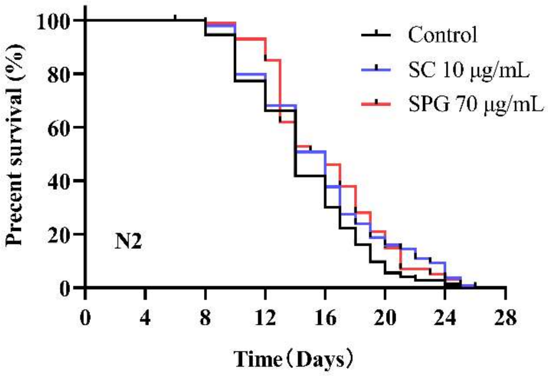

Figure S2. Effect of SC and SPG on the lifespan of *C. elegans*. During the reproductive period, worms were scored daily and transferred to new treatment dishes every other day. The survivals were recorded every other day, until all of the worms died. There were at least three independent biological replicates (n ≥ 50). Data were expressed as the mean ± SD (n = 3).

Table S1. Primer sequences. This table includes all primers used in RT-qPCR experiments.

Gene	Gene ID	Direction	Primer sequences (5'-3')
Caenorhabditis elegans actin-1	179535	Forward	TACTCTTTCACCACCACCGC
		Reverse	ACGGTGATGACTTGTCCGTC
Caenorhabditis elegans daf-2	175410	Forward	TGAAAGCGAAGCAGCGAGAAGG
		Reverse	CGTCCGAACTTCCGCATCACTC
Caenorhabditis elegans daf-16	172981	Forward	CGGGAGAGAGGGACACGCTTC
		Reverse	ACGGAATTGCTCAGCCACCATG
Caenorhabditis elegans age-1	174762	Forward	CGCCACGGCAACATCCTCAG
		Reverse	GGCTGCTCAATCGCCAACTCC
Caenorhabditis elegans sod-3	181748	Forward	CATTGTTTCAGCGCGACTTCGG
		Reverse	TCCCCAGCCAGAGCCTTGAAC
Caenorhabditis elegans mev-1	260040	Forward	GCCCAATCGCTCCACATCTCAC
		Reverse	CGAGAAGGGTTCCGGCCATTAC
Caenorhabditis elegans clk-1	175729	Forward	AGTGTGGCTGCTTATGCTCTCG
		Reverse	GCTGAACCGACACCTGCAAGG
Caenorhabditis elegans skn-1	177343	Forward	AAACGAAGACGAAGACAGTGC
		Reverse	TTGAGGTGTTGGACGATGGTG
Caenorhabditis elegans gst-4	177886	Forward	GCTCAATGTGCCTTACGAGG
		Reverse	GCAGTTTTTCCAGCGAGTCC

Table S2. Effect of SC and SPG on the lifespan of *C. elegans.* (mean ± SD, n = 3)

Group	Mean Lifespan (Days)	Maximum Lifespan (Days)	Number	Mean fold Increase % [a]
Control	16.27 ± 1.34	23.50 ± 2.12	115/150	-
SC (10 μg/mL)	15.47 ± 0.64	25.67 ± 0.58	117/150	-4.92
SPG(70 μg/mL)	16.06 ± 0.25	25.00 ± 0.00	116/150	-1.29

$^{*}P < 0.05$; $^{**}P < 0.01$; $^{***}P < 0.001$. [a] Percentage of mean fold increase is relative to the control.
